# Arl13b regulates early endocytic vesicle trafficking

**DOI:** 10.1186/2046-2530-1-S1-O7

**Published:** 2012-11-16

**Authors:** DC Barral, S Garg, C Casalou, G Watts, JS Ramalho, VW Hsu, MB Brenner

**Affiliations:** 1 CEDOC - Faculdade de Ciências Médicas da Universidade Nova de Lisboa, Portugal; 2 Brigham and Women's Hospital, Harvard Medical School, USA

## 

We attempted to find new proteins involved in the regulation of the endocytic recycling pathway used by CD1a, a MHC Class I-like lipid antigen-presenting molecule that follows an endocytic recycling pathway similar to that used by MHC Class I and other cargo internalized independently of clathrin. For that, a shRNA library comprising the main families of proteins known to be involved in membrane trafficking was used to screen HeLa:CD1a cells for changes in CD1a surface expression that could reflect intracellular trafficking defects. Our finding that Arl13b silencing leads to a decrease in CD1a surface expression and function, as well as a delay in CD1a recycling, suggests that this ciliary protein is involved in the regulation of endocytic recycling trafficking. Moreover, Arl13b silencing caused the clustering of early endosomes and the accumulation in this organelle of recycling cargo, such as transferrin, and also cargo destined for late endosomes and lysosomes, such as dextran. We also found Arl13b to colocalize with Arf6 mutants that lead to an accumulation of clathrin-independent recycling cargo and in recycling tubules labeled by Rab22a. Together, these results indicate that Arl13b regulates a sorting step from the early/sorting endosome. Surprisingly, we found Arl13b to colocalize with actin filaments and to immunoprecipitate with actin, which brings mechanistic insight into the function of this Arl protein. Thus, our results indicate a previously unidentified role for Arl13b in the endocytic recycling pathway and suggest a link between Arl13b function and the actin cytoskeleton.

